# AI Prediction of Brain Signals for Human Gait Using BCI Device and FBG Based Sensorial Platform for Plantar Pressure Measurements

**DOI:** 10.3390/s22083085

**Published:** 2022-04-18

**Authors:** Asad Muhammad Butt, Hassan Alsaffar, Muhannad Alshareef, Khurram Karim Qureshi

**Affiliations:** 1 College of Chemicals & Materials, King Fahd University of Petroleum & Minerals, Dhahran 31261, Saudi Arabia; 2 Electrical Engineering Department, King Fahd University of Petroleum & Minerals, Dhahran 31261, Saudi Arabia; s201582450@kfupm.edu.sa (H.A.); s201467960@kfupm.edu.sa (M.A.); 3 Physics Department, King Fahd University of Petroleum & Minerals, Dhahran 31261, Saudi Arabia; 4 Optical Communications and Sensors Laboratory (OCSL), Electrical Engineering Department, King Fahd University of Petroleum & Minerals, Dhahran 31261, Saudi Arabia; kqureshi@kfupm.edu.sa; 5 Center for Communication Systems & Sensing, King Fahd University of Petroleum & Minerals, Dhahran 31261, Saudi Arabia

**Keywords:** brain computer interface fiber Bragg grating, healthcare, artificial intelligence, plantar pressure

## Abstract

Artificial intelligence (AI) in developing modern solutions for biomedical problems such as the prediction of human gait for human rehabilitation is gaining ground. An attempt was made to use plantar pressure information through fiber Bragg grating (FBG) sensors mounted on an in-sole, in tandem with a brain-computer interface (BCI) device to predict brain signals corresponding to sitting, standing and walking postures of a person. Posture classification was attained with an accuracy range between 87–93% from FBG and BCI signals using machine learning models such as K-nearest neighbor (KNN), logistic regression (LR), support vector machine (SVM), and naïve Bayes (NB). These models were used to identify electrodes responding to sitting, standing and walking activities of four users from a 16 channel BCI device. Six electrode positions based on the 10–20 system for electroencephalography (EEG) were identified as the most sensitive to plantar activities and found to be consistent with clinical investigations of the sensorimotor cortex during foot movement. A prediction of brain EEG corresponding to given FBG data with lowest mean square error (MSE) values (0.065–0.109) was made with the selection of a long-short term memory (LSTM) machine learning model when compared to the recurrent neural network (RNN) and gated recurrent unit (GRU) models.

## 1. Introduction

**Figure 1:**
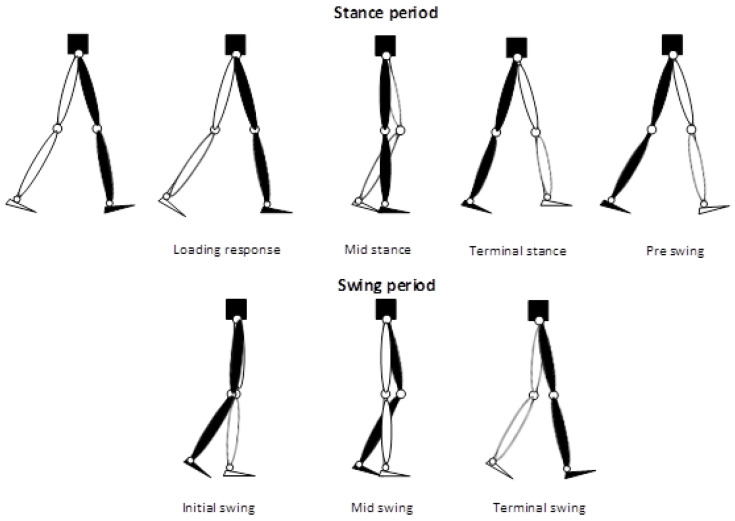
A typical human gait cycle.

Human gait and other clinical investigations related to human biomechanics are essential to the well-being of patients suffering from impediments in locomotion. This can arise due to numerous circumstances such as injuries, neurological disorders [1] and adaptation to prosthetic devices in case of amputation. Such investigations are not only useful in human rehabilitation but also support the understanding for developing robots that can mimic human motions and maintain gait stability while performing various human like activities such as sitting, standing, walking, running and jumping. The human gait is illustrated in Figure 1, where successive actions to support walking while maintaining contact of the foot with the ground are shown. Other human postures such as sitting and standing are also established using foot contact with the ground. The perception of these postures in the brain through pressure profiles on the sole (plantar pressure) developed during various contact scenarios enables stable gait.

Considerable research has been done in the area of plantar pressure investigations [2,3,4,5]. The human foot sole has plantar nerves that carry information to the brain motor cortex, where it is perceived and interpreted for an adequate response. In the case of foot amputation, the sensory loss disables the patient’s perception of foot contact and develops psychological trauma [6]. A large number of patients who undergo amputation in the lower extremities are those with vascular diseases such as diabetes [7]. The lower extremity loss could mean the loss of a foot or the entire leg. The annual number of amputees in the lower extremities in the US alone is around 185,000 as determined by the Amputee Coalition of the USA. In the Middle East and North Africa (MENA) region, Saudi Arabia has the highest rate of lower limb amputation owing to diabetes [8]. To improve the quality of life of the amputees and alleviate the psychological trauma that the patients suffer, lifelike prosthetic attachment to the body plays an important role.

The current state of the art of prosthetic devices [9,10,11] has created numerous opportunities for researchers to venture into areas where using miniature sensors that can be embedded into in-soles can help determine plantar pressures [1,12,13]. One such sensor is the fiber Bragg grating (FBG), which in recent times has found its applications in novel medical applications [14,15,16]. FBGs are also being implemented in plantar pressure measurement systems [1,17]. The advantages of FBGs lie in their miniature size, sensitivity, immunity to electromagnetic interference (EMI) and resistance to harsh conditions once applied with adequate protective coating [18].

Artificial intelligence (AI) has taken the research in biomedical applications to new heights by providing highly accurate diagnosis and prognosis in the presence of sufficient patient data [19]. The involvement of AI in human gait prediction [20,21] allows lesser efforts and accurate diagnostics for the identification of the patient’s disease(s) and suggestions for corrective actions. Other than plantar pressure information, brain electroencephalography (EEG) also provides a valuable insight into the patient’s perception of touch (in this case, touch of the foot). BCI devices have been used in recent research to study and monitor brain activity using EEG signals [22]. The EEG signal analysis has been widely studied in the last decade. Many machine learning methods, such as SVMs, KNNs [23], artificial neural networks (ANNs), deep neural networks (DNNs) [24], etc. have been presented for EEG signal processing. In addition, many new signal processing techniques such as [25,26] have been used for EEG signal analysis. Companies such as Neuralink are unraveling the mysteries of the human mind through implantable brain computer interfaces.

Haptic classifications [27] are also important but the understanding of primary contact scenarios through plantar pressure variation is vital. In case of sensory loss [28], the brain motor cortex retains the memory of the pre-amputation sensorial feedback [29] and if understood properly, can be reconstructed through the plantar pressure of the able foot. Research into a comprehensive product that can enable patients to regain sensory feelings through plantar pressure measurements, BCI and the use of AI will have a substantial impact on society and provide opportunities for technology entrepreneurs to venture into biomedical applications.

Thus, an investigation was conducted to design a process for estimating EEG signals through plantar pressure using FBG sensors. Plantar pressure and FBG data were collected from four men, aged 23 to 24 years. The participants filled out a consent form agreeing to provide their clinical data. The experiments were designed in two parts. In the first part, two participants wore a BCI device that is head mounted and performed actions of a. sitting, b. standing, and c. walking. This experiment was conducted to perform the classification of postures from the incoming data of the BCI device. The classification of activities is followed by a second part of the experiment, where four participants perform the sitting, standing and walking activities to collect enough data samples to run a machine learning model for predicting the brain EEG signal in a specific posture. The four participants wore an FBG mounted insole with FBGs located at three high pressure points in the plantar region, in addition to the head mounted BCI device for recording the EEG signals. 

The paper is organized in the following manner: Section 1 introduces the topic and the necessity of the research in the specific domain. Section 2 discusses the sensors used, their placement and the importance of the BCI for this research. Section 3 explains the test setup focusing on the devices used and data collection from the FBG sensors and the BCI. Section 4 details the AI implementation with insight into experiment design, preparation for data recording and utilization of various machine learning models for the two parts of the experiment (as discussed earlier), while providing results and discussion along with the conclusion in Section 5. Finally, Section 6 provides recommendations for future work.

## 2. FBG Sensors and BCI Device

Two different types of sensors were used in this work. FBG sensors to collect plantar pressure data and electrodes mounted on the headset of a BCI device to collect the EEG signals. A description of the FBG sensor and the BCI device is explained further.

### 2.1. FBG Sensor

**Figure 2:**
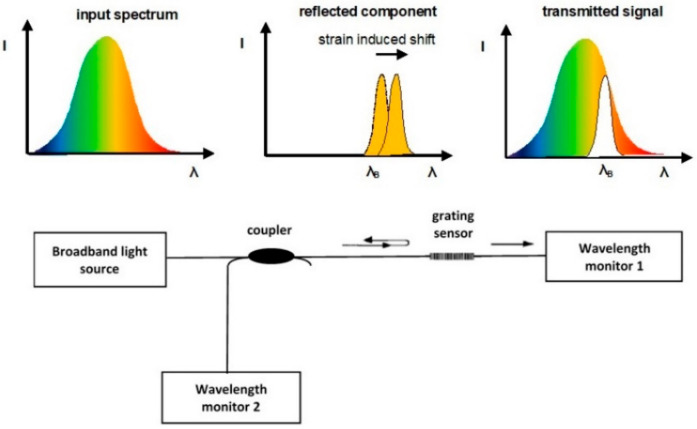
Working principle of FBG sensors and associated inspection scheme.

Fiber Bragg grating (FBG) sensors are based on optical fiber technology, where a specific region in the fiber (usually 10–20 mm along fiber length) is etched by ultraviolet (UV) radiation to produce gratings with a specific period. When light passes through the fiber, the grating period provides a distinctive reflective wavelength signature of the fiber in the etched section under Bragg’s law and responds to changes in pressure and temperature of the fiber with a shifting wavelength on an optical interrogator display as shown in Figure 2.

The FBGs are small in size (250 μm diameter) and sometimes coated with the protective layer to protect from harsh environments, as shown in various characterization studies [30].

### 2.2. Brain Computer Interface (BCI) Device

**Figure 3:**
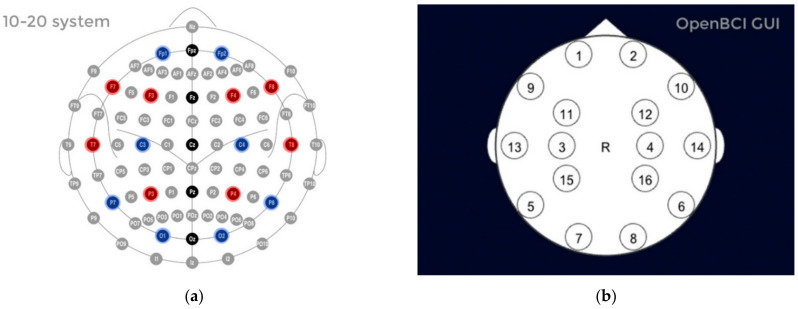
(**a**) 10–20 system for EEG monitoring on the scalp; (**b**) 16-electrode positioning by the Open BCI headset.

**Figure 4:**
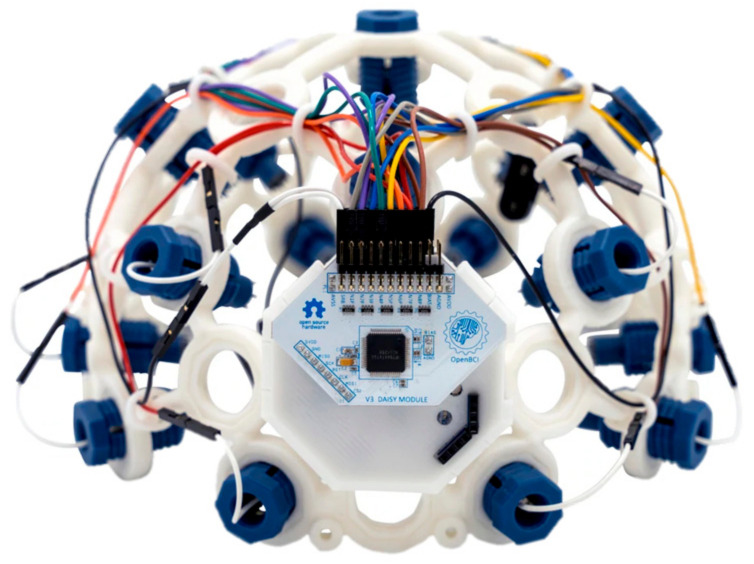
Open BCI Ultracortex Mark IV Headset with on-board biosensing circuit.

**Figure 5:**
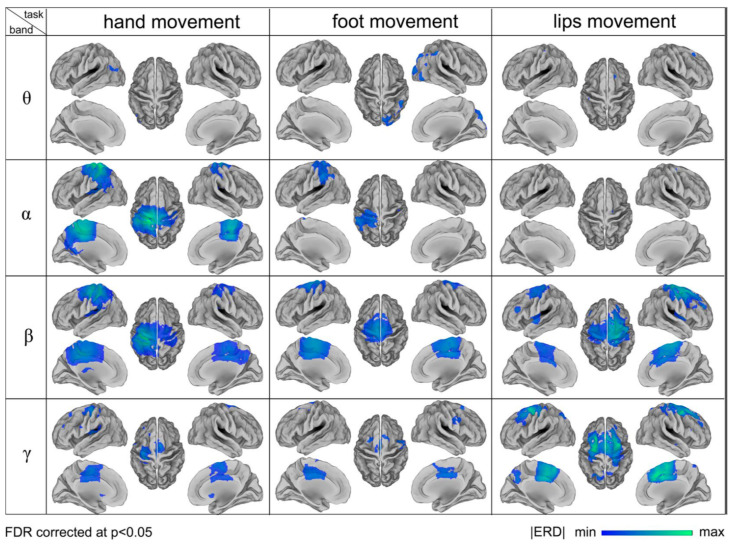
Comparison of brain activity monitored during hand, foot, and lip movement [35].

Brain mapping of various human activities can be produced through magnetic resonance imaging (MRI) [29,31], computer tomography (CT) scans and BCI devices [32,33] that pick up electrical impulses generated by the neuron firing in the brain through electrodes positioned over the head scalp of the patient. Many modern BCI devices use the standard 10–20 electrode placement for EEG examination. The 10–20 system or International 10–20 system is an internationally recognized method to describe and apply the location of scalp electrodes as shown in Figure 3. The BCI device used in current research is produced by OpenBCI, which creates tools for biosensing and neuroscience. The particular BCI contains 16 electrodes providing 16 channels with Cyton + Daisy biosensing board and an Ultracortex Mark IV EEG headset that comes pre-assembled as shown in Figure 4. The brain activity during foot movement has been previously cited in [34,35] and is shown in Figure 5.

The objective of this research is to study brain signals and plantar pressure, analyze, and predict them in order to facilitate a healthy gait cycle for patients, including the following:

1. Designing an automated process that can improve on gait analysis data and provide valuable insight for the development of smart prosthetics in the future.
2. Incorporating patient’s feedback using plantar pressure sensors and brain signals for correction and calibration.
3. Creating a classification model that identifies brain activity related to plantar pressure profiles and predicts brain activities signals based on monitored plantar pressure.

## 3. Experimental Setup

**Figure 6:**
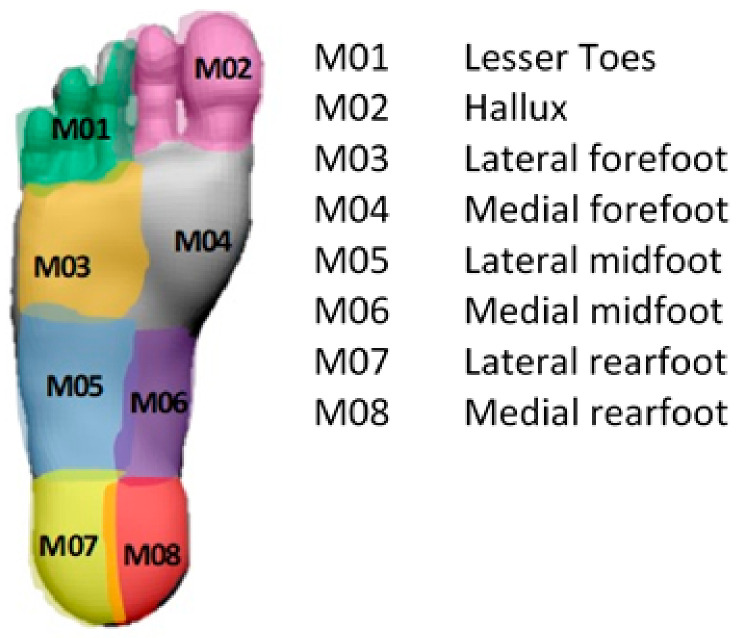
Foot plantar area divided into different regions.

This section provides the details of the experimental setup utilized in the study. Before advancing into data collection, it was important to identify the sensor positioning. It is of critical importance to map an accurate ground contact scenario through the measurement of plantar pressure in key regions of the foot sole. The foot sole can be divided into numerous regions as shown in Figure 6.

**Figure 7:**
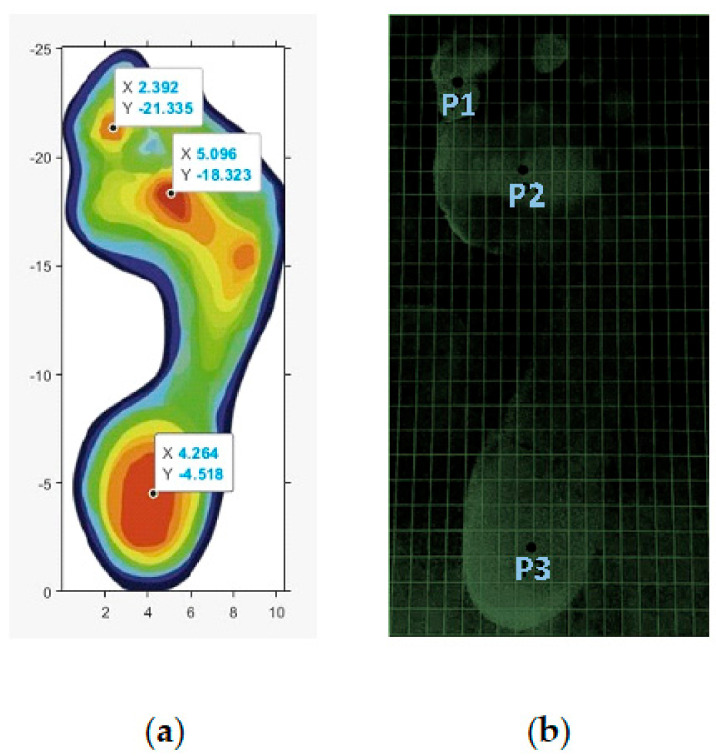
(**a**) Sensor location identified with MATLAB code; (**b**) foot pressure identified with markings of the ink pad.

Many researchers have introduced piezoresistive sensors at eight different locations [36], photo-resistive at six [37], and FBG sensors at five locations on the in-sole [38]. Our work incorporates three stand-alone FBG sensors placed at (i) the juncture of M02 and M04, (ii) the juncture of M03 and M04, and (iii) the juncture of M07 and M08 as shown in Figure 7a. The selection of these points come from previous clinical investigations of young adults [39]. The pressure map created with an ink pad is shown in Figure 7b for each participant. The clinical data correlation to the information provided by the participants such as age, weight, height, and foot size is shown in Table 1. A MATLAB script was prepared to locate the positions of sensor placement for all of the four participants. Later on, FBG data were collected from four sets of locations slightly different for each individual to capture maximum pressure values based on the variation in Table 1.

**Figure 8:**
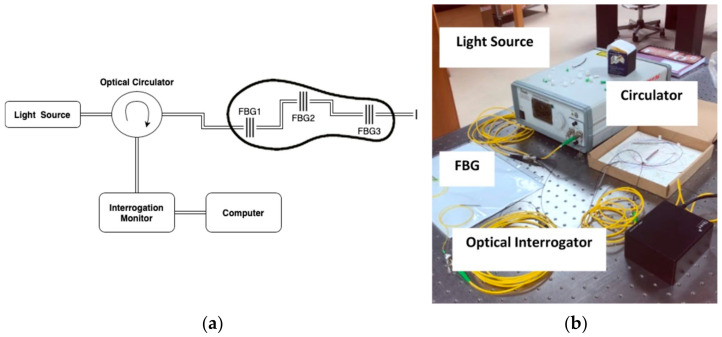
(**a**) Schematics of the experimental setup; (**b**) experimental setup at the lab.

The experimental setup consists of three FBG sensors connected to a broadband light source (1510–1590 nm) and a compact optical interrogator IMON USB 512 produced by IBSEN Photonics. All the devices are connected through an optical circulator, as shown in Figure 8.

**Figure 9:**
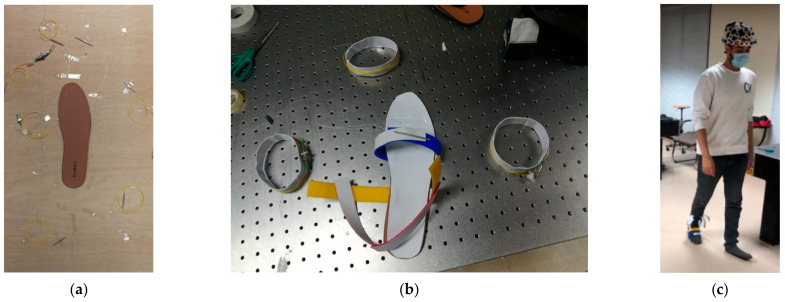
(**a**) Three FBGs installed on the insole; (**b**) sandal prepared for multi-user use; (**c**) a participant walking while wearing the BCI headset and the FBG installed insole.

**Figure 10:**
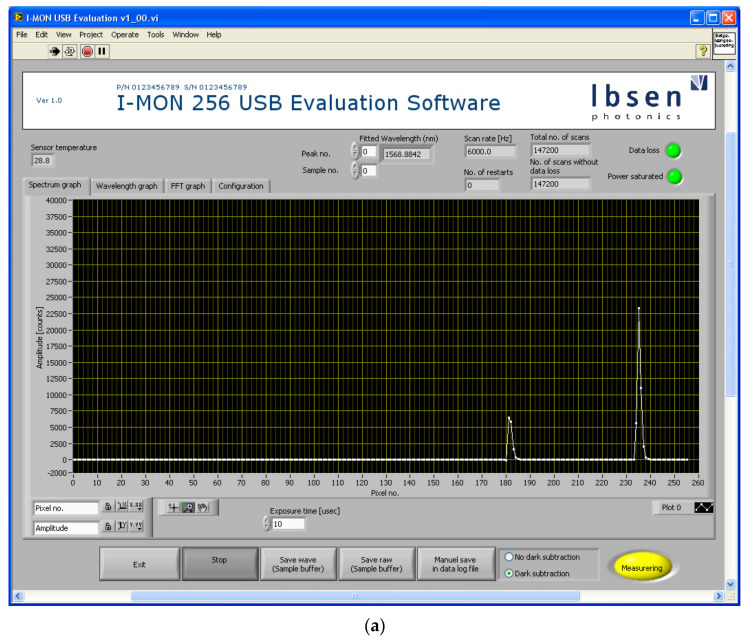

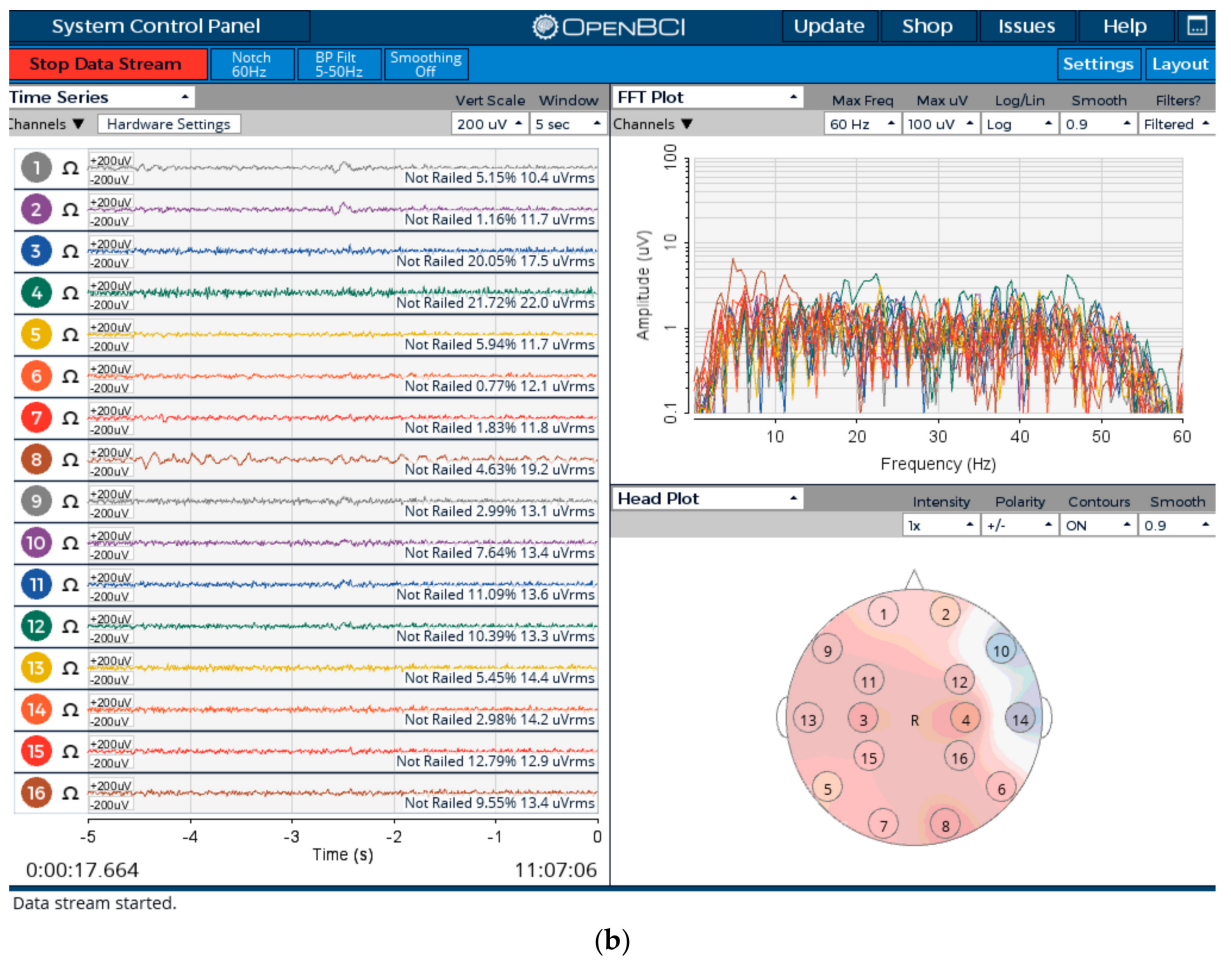
(**a**) Optical interrogator GUI module; (**b**) open BCI system control panel GUI 16 channel collection.

The FBG sensors were placed on a 5 mm thick polyethylene foam insole, as shown in Figure 9a. The FBGs were located at customized locations based on the user’s foot size, weight, and height. The in-sole prototype equipped with 3 × FBGs were glued in place in a wearable sandal arrangement with a velcro strap-on for multi-user design, as shown in Figure 9b. One of the participants can be seen in Figure 9c wearing the BCI headset and the FBG installed insole. Data visualization is available through two graphical user interfaces (GUIs), (i) an Ibsen Evaluation Software for FBG wavelength monitoring and (ii) an OpenBCI System Control Panel as shown in Figure 10. In Figure 10a, the GUI for the FBG interrogator allows the user to monitor and plot the connected FBGs identified by their signature wavelengths. While selecting FBGs, it is important that the wavelength shift indicative of the FBG response to applied pressure does not overlap with other FBG wavelength shifts. The optical interrogator registers the peaks for each FBG by its corresponding wavelength signature. In our case, the three FBGs had wavelength values of 1535.068 nm, 1539.966 nm, and 1545.341 nm corresponding to midfoot, heel, and toe areas of the foot region. Figure 10b shows the GUI for the BCI device, where brain activity registered by the 16 electrode channels are shown separately in the left part of the GUI and collectively in the right top corner (Amplitude vs. Frequency-FFT plot). The amplitude values, i.e., microvolt (μV) readings, were used as the data to interpret the brain’s response to various foot movements during posture change and walking action. The bottom right corner of the GUI window shows the head plot indicating the activity region in the brain corresponding to various body actions of the user and the 16-electrode positioning on the user’s head.

## 4. AI Implementation and Results

In this section, various classification and machine learning (ML) models were implemented to classify and predict BCI signals from a set of experiments involving participants wearing the BCI device and the FBG installed insole. The experiments were performed in two parts, namely Part I—BCI Classification and Part II—BCI Prediction. As explained earlier, the purpose of the first part of the experiment was to identify the electrodes on the BCI headset that are more responsive to foot movement. Later in the second part, BCI signals were predicted against a random plantar pressure information. The AI was implemented using Python version 3.10.4 (open source software) and pre-processing of the data was performed in MATLAB version 2021b. For clarity, the experiments and their AI implementation are presented in two parts.

### 4.1. Part I—BCI Classification

In this part, the collection of EEG data was performed from the head mounted BCI device. The data was collected from two participants. The purpose was to perform a classification of the incoming data from the BCI device in the a. sitting, b. standing, and c. walking postures. This helped identify the BCI signals directly associated with the foot movement with a reduced number of channels compared to the original 16. 

#### 4.1.1. BCI Classification Experiment

**Figure 11:**
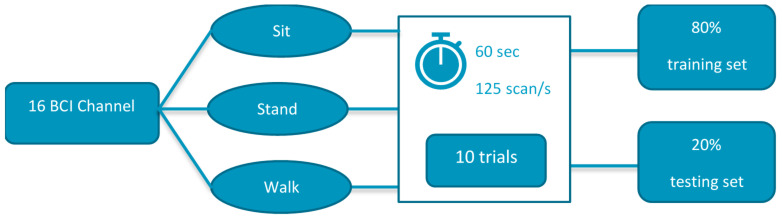
Schematic for part I of the experiment (BCI Classification).

The experimental setup consisted of wearing the BCI device and performing the three gait positions, where the participant sits in a chair of height 50 cm and rests his feet on the ground. For standing, while maintaining a good posture, the participant stands with minimal movement. Finally, the walking gait is recorded by walking in a straight path for 60 s. The schematic for the experiment is shown in Figure 11.

**Figure 12:**
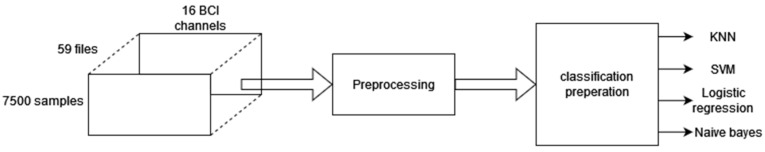
Signal flow from data collection to classification.

Before creating machine learning models, the data were split randomly into two datasets. The first set contains 80% of the data set and is called a training set in which the classification technique extracts information to build a model. The second set was used to test the model extracted from the training set, called the testing set. Four classification models, namely 1. K-nearest neighbor (KNN), 2. support vector machine (SVM), 3. logistic regression (LR), and 4. naïve Bayes (NB) were used. The dataset was preprocessed before using the classification models. The classification result also helped to identify the key electrode positions responsible for the foot activity. A reduced number of BCI channels eased the burden on correlation during the second part of the experiment. The process of classification is summarized in Figure 12.

The classification dataset was recorded from two participants for three different gait positions: sitting, standing, and walking. The brain activity was collected for each participant for ten trials, each for 60 s in the three gait positions. Each trial has data from 16 electrodes sensing the brain activity, which provides 16 signals in each trial. Hence, the total number of trials was 59 (one standing file got corrupted). Each signal has 7500 data points since the sampling frequency was 125 Hz. Furthermore, the data were reorganized such that all the signals from all trials for one electrode were in one data file, resulting in 16 data files. Hence, the brain activity signal was used as the only feature for the classification model. The output variable of the dataset is the gait position; therefore, these outputs were encoded as 0 for sitting, 1 for standing, and 2 for walking.

#### 4.1.2. BCI Data Pre-Processing

The dataset was preprocessed by removing the first and last 5 s for stability purposes, detrended (removing the mean of each channel), normalized using the z-score method, and low passed at 60 Hz to remove the 60 Hz main noise. From the BCI data, the difference between sitting and walking is not apparent in some channels, which means that brain signals do not have a one-to-one mapping to sensory perception, making it harder to classify or use machine learning models since the signal is a composition of different factors; however, several models can be used to obtain decent accuracy, e.g., K-NN and Naïve Bayes, only for the channels that are relatively sensitive to plantar pressure.

**Figure 13:**
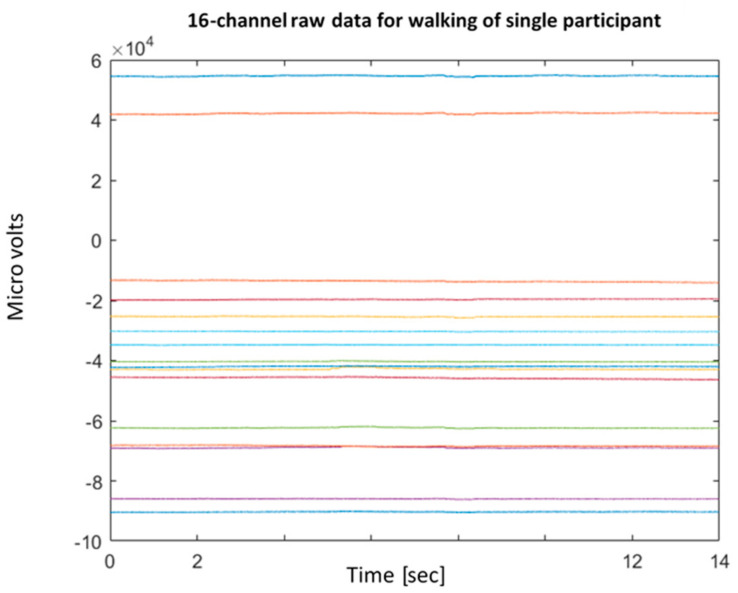
Raw EEG data of the 16 channels over 14 s during walking of a single participant.

A sample of 14 s EEG data was investigated for the three postures for each participant as shown in Figure 13. Two main methods were used to denoise the signal. Method 1 applied *normalization* followed by *detrending* and application of a *low pass filter*. In method 2, detrending is applied using wavelet decomposition. These methods are a part of preprocessing data before their use in machine learning models.

**Figure 14:**
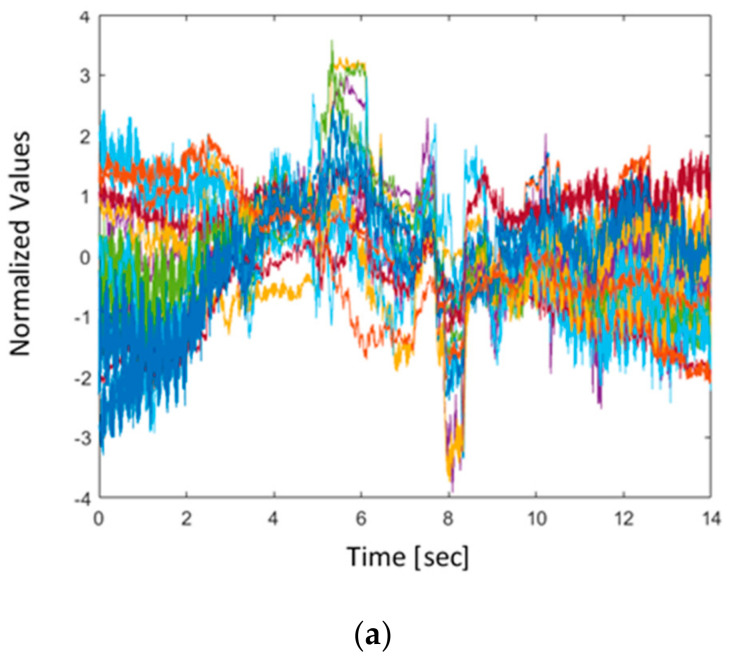

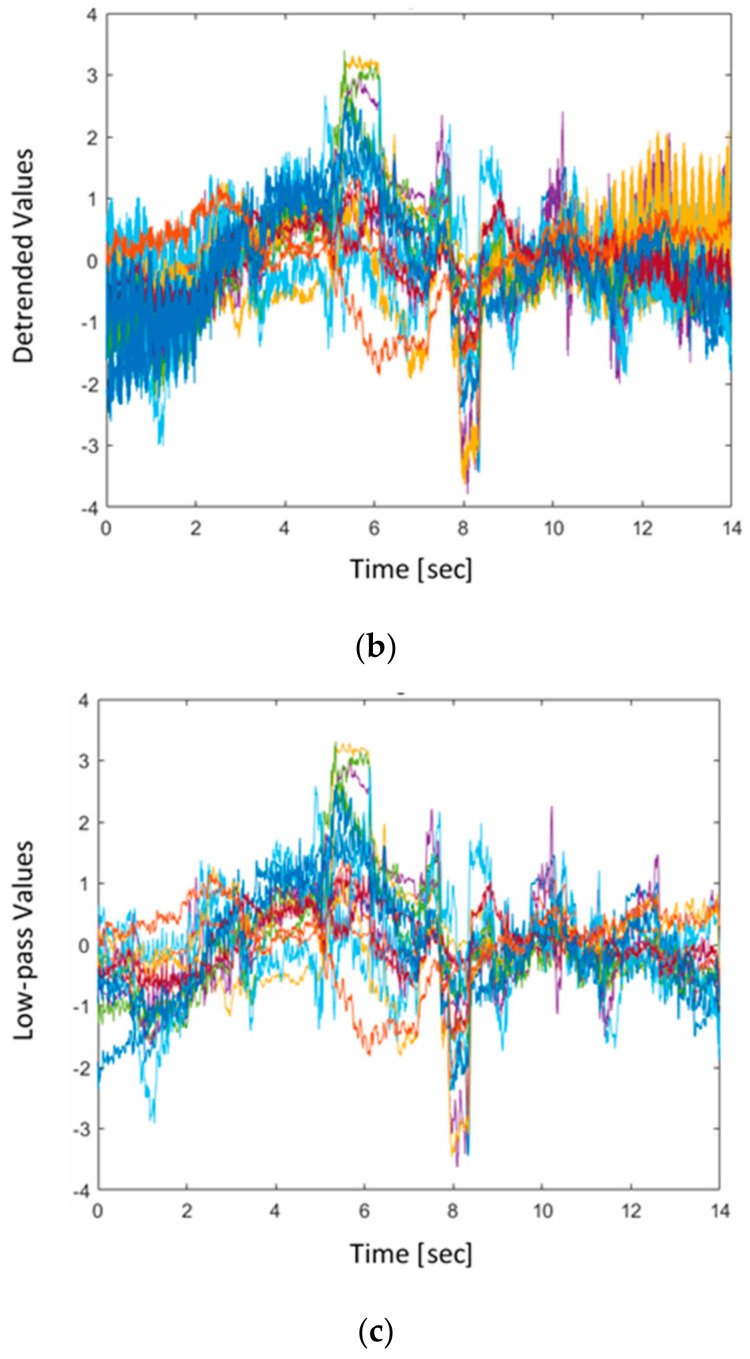
(**a**) Normalized channels using the z-score method; (**b**) detrended signal by removing the mean; and (**c**) low-passed signals at 60 Hz.

As an example, the BCI EEG 16 channel data showing 14 s of walking activity before and after preprocessing by method 1 can be seen in Figure 13 and Figure 14. *Normalizing* involved normalizing the signals using the z-score so the magnitude difference between the tests would be negligible. This also results in removing the DC offset of the channels. Instead of using a high-pass filter at 0.1 Hz, *detrending* removes the mean of each channel. Detrending was used since it can improve the accuracy of machine learning, as in case study [40]. Although not perfect, this was better than introducing artifacts caused by interpolation detrending. Finally, a *low-pass filter* was applied to remove the line noise at 60 Hz.

Method 2 using the *wavelet decomposition* allowed detrending the signal in a much more effective way; as seen in [40], a 4-level sym4 was used instead of db4 due to better performance according to [41], while [42] discussed the use of wavelet transforms for EEG classification.

**Figure 15:**
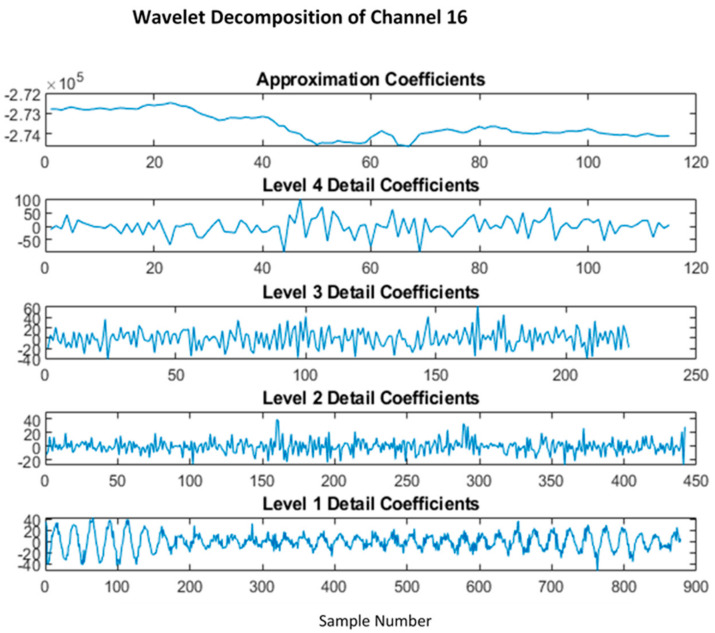
4-level wavelet decomposition using sym4 wavelet.

**Figure 16:**
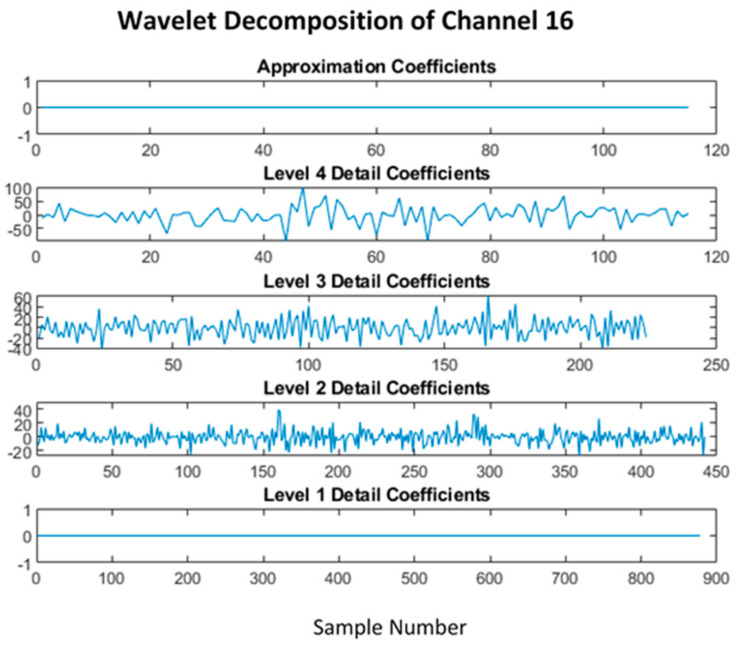
Wavelet decomposition with both approximation and level 1 detail removed.

**Figure 17:**
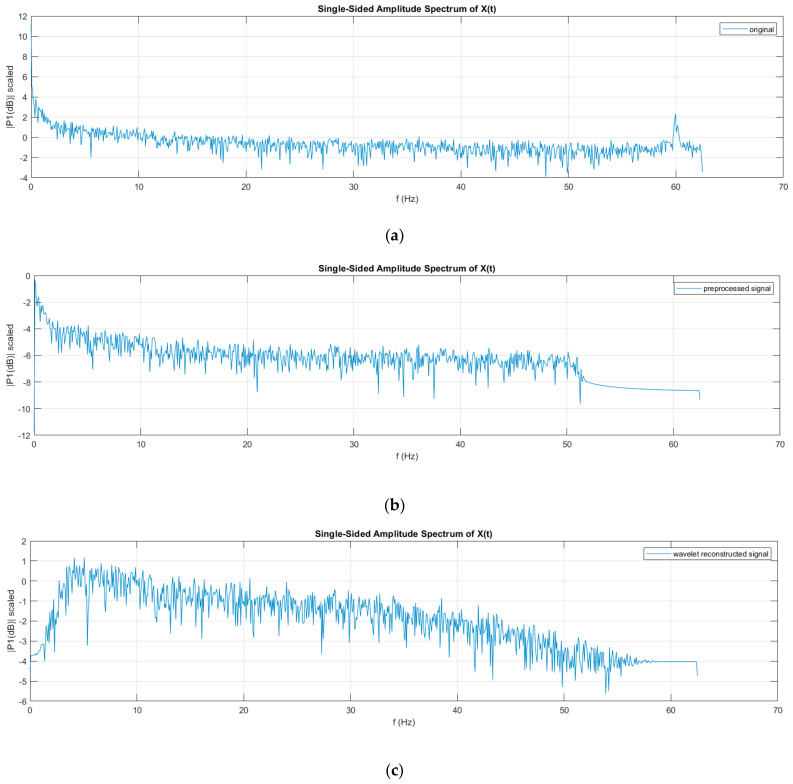
Fourier transform comparison of channel 16 (**a**) before; (**b**) after pre-processing; and (**c**) after wavelet processing.

The level 1 detailed coefficient contains the 60 Hz line noise as shown in Figure 15, while the approximation contains the low-frequency trend of the signal, as discussed in the normal pre-processing. The trend and the line noise were removed by removing both these coefficients, as shown in Figure 16. Fourier transform comparison of channel 16 before and after wavelet processing is shown in Figure 17.

#### 4.1.3. Classification Models

For the classification models, k-fold cross-validation was used on the training set, where the training set was divided into k groups. The data was trained using k-1 groups, and the remaining group was used to test the performance of the model before trying it on the test set. The test set was only allowed to be used once after trying to obtain the best model possible using k-fold cross-validation. The value of ‘k’ was chosen to be 4.

After encoding the data into three classes, and splitting the data into training and testing, four classification algorithms were used in our study, which were provided by the *Sklearn* library. Each electrode datafile had its own classifier using different techniques.

##### K-Nearest Neighborhood (KNN)

This method compares a test tuple and similar training tuples, which are described by n features. In n-dimensional space, each training tuple corresponds to a point. Thus, all training tuples are stocked in n-dimensional pattern space. The classifier searches the pattern for k training tuples that are closest to the unknown tuple, which determines the class of the unknown tuple. K training tuples are called k nearest neighbors [43].

In our models, the classifiers were optimized based on the distance equation, the number of neighbors considered, and the weight of each sample in the space. Four methods for distance calculation were considered: Euclidean, Manhattan, Chebyshev, and Minkowski. All three variables were chosen to obtain the highest accuracy model for each channel’s classifier. Hence, the number of k neighbors differs for each channel classifier. Also, a few of the classifiers had the best performance when the closest neighbors have a higher influence on the classification decision. In contrast, others performed better when all neighbors had the same influence on the classification output. Finally, most of the classifiers used the Chebyshev equation for distance to obtain the best performance.

##### Support Vector Machine (SVM)

A support vector machine is used to classify linear and non-linear data. Training data can be transformed into higher dimensions to be able to find an optimal linear hyperplane in the new dimension using non-linear mapping. A hyperplane separates the data of each class from the data of the other classes. Support vectors and margins are the main methods to find this hyperplane [44].

The classifiers were optimized based on the regularization parameter (slack variable C), kernel equation, and kernel coefficient, i.e., gamma. The regularization parameter is a control on the fitting parameters that provides a penalty on the cost function to reduce overfitting. It ranged from 10^−10^ to 10^4^ and each classifier had a different value to maximize the performance. This means that the slack variable C determines how many samples can be inside the margin and wrongly classified. Moreover, four types of kernel equations were used: linear, polynomial, radius bias function (RBF), and sigmoid. The kernel equation was determined by the accuracy of the classifier. The gamma determines the decision region (the radius) of the vector, and it does not apply to the linear kernel. However, it varied from 10^−15^ to 10^4^ for polynomial, RBF, and sigmoid kernels. Finally, the value of each variable was determined by the best-fitting models for each electrode classifier to obtain the highest accuracy, e.g., for channel 2: C = 1 and gamma = 0.01 and for channel 6: C = 100 and gamma = 0.01.

##### Logistic Regression (LR)

Another powerful supervised machine learning algorithm for classification is logistic regression [45]. It models a discrete output given a continuous input variable. It is usually used for a binary output, but it can be used for multinomial outputs. It is simply a linear regression model with a logistic function that bounds the output between 0 and 1, and it does not require a linear relationship between inputs and outputs. This type of multi-class logistic regression or multinomial regression is known as SoftMax regression. 

The slack variable C was the main hyperparameter obtained to maximize the performance of the classifiers. Another possible parameter to optimize was the solver (the algorithm used to optimize the model fitting). However, due to the nature of the dataset, only the linear solver was used.

##### Naïve Bayes (NB)

This is a classification method based on Bayes’ theorem with an additional assumption of data independence, which means that it assumes the features are not related to each other. Bayes’ theorem says that the posterior probability of a class is equal to the likelihood times the class prior probability over the predictor prior probability. Furthermore, there are different assumptions about the likelihood such as Gaussian, multinomial, and Bernoulli [46]. However, only Gaussian likelihood was considered in our model. The Gaussian has variance smoothing as a hyperparameter, which was determined by the highest accuracy for each classifier.

#### 4.1.4. Performance Measure

**Figure 18:**
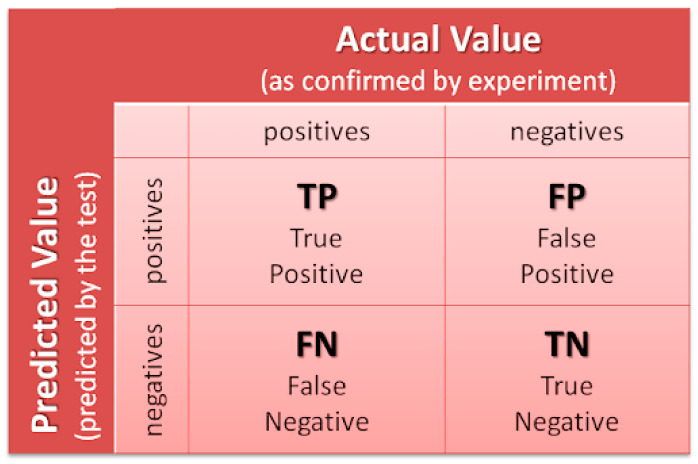
Confusion Matrix.

The performance measure for the first experiment is accuracy and balanced accuracy. These two measures compare the classifier’s ability to choose a suitable class. The accuracy is the ratio between the number of the current prediction to the total number of predictions and is given by Equation (1):

(1) $$\mathrm{Accuracy}=\frac{\mathrm{TP}+\mathrm{TN}}{\mathrm{TP}+\mathrm{FP}+\mathrm{TN}+\mathrm{FN}}$$

where TP, TN, FP, and FN are explained in Figure 18. The balanced accuracy is a metric used when the classes are imbalanced since each class has an equal contribution to the result. Due to the low number of samples, and randomness of choosing the Train, Val, and Test sets, the balanced accuracy provides more reliable results than the accuracy and is calculated by Equation (2):

(2) $$\mathrm{Balanced}\;\mathrm{Accuracy}=\frac{\mathrm{Sensitivity}+\mathrm{Specificity}}{2}$$

where sensitivity is given by Equation (3):

(3) $$\mathrm{Sensitivity}=\frac{\mathrm{TP}}{\mathrm{TP}+\mathrm{FN}}$$

and specificity is given by Equation (4):

(4) $$\mathrm{Specificity}=\frac{\mathrm{TN}}{\mathrm{TN}+\mathrm{FP}}$$

The classification model was run on 59 files for raw sampled data and preprocessed BCI data (one of the 60 files was corrupted), and Table 2 provides the accuracy results for each channel and classification model utilized. In addition, BCI channels directly correlated to plantar foot pressure were acquired as a result of this classification and included in the machine learning model.

**Figure 19:**
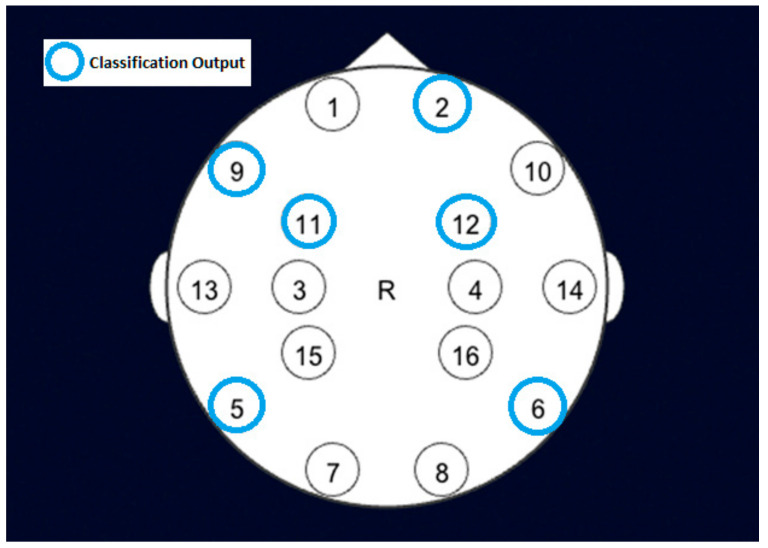
BCI Channels (EEGs) and Classification Output.

The channels with the best accuracy for the results shown in Table 2, are 2, 5, 6, and 9 by K-NN (K-nearest neighbors) and 6, 9, 11, and 12 by naive Bayes, as shown in Figure 19. The K-NN model performed better on the raw data than the preprocessed data because it depends on the distance between the point and its neighbors. The distance between points in the raw data is untouched, while preprocessed data distances are changed because of detrending. The gait posture influences channels 2, 5, 6, and 9 since they have better accuracy than the rest of the channels and must be considered in the second part of the experiment. Moreover, detrending the data removes the average value of each signal. The data variance plays a prominent role in the NB classifier on the preprocessed data. 

On the other hand, the data affected by the gait posture has smaller variance. Thus, it has higher NB classifier accuracy as in channels 6, 9, 11, and 12. Therefore, the raw and preprocessed data results were carried to part II since they provided the channels that are sensitive to gait posture from different perspectives to ensure all possibilities.

For verification purposes, the findings of the classification model were compared to other literature. For example, it agrees with the recorded brain activity by magnetic resonance imaging (MRI) of participants who were supine and had a force applied on their sole [47].

### 4.2. Part II—BCI Prediction

In this part, the plantar pressure data collection from the FBG mounted insole was performed with FBGs located at three high-pressure points in the plantar region. The data were in addition to the BCI data simultaneously recorded from four participants while performing sitting, standing, and walking activities to collect enough data samples to run a machine learning model and predict the brain EEG signal in a specific posture.

#### 4.2.1. BCI Prediction Experiment

**Figure 20:**
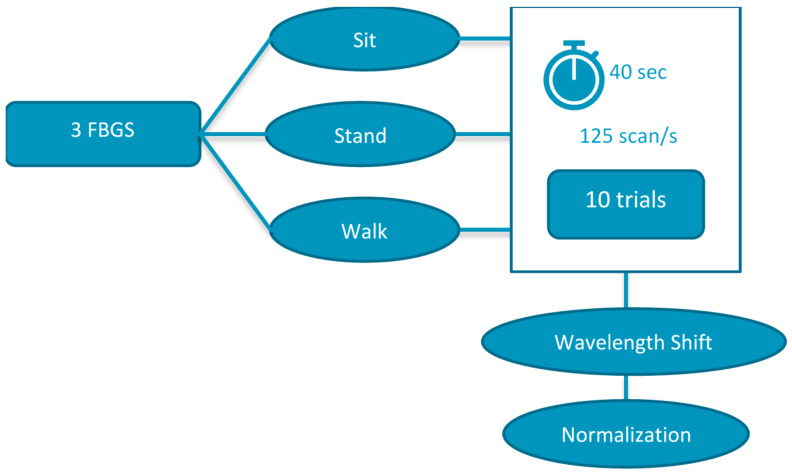
Schematic for part II of the experiment with FBG data collection (BCI Prediction).

The number of trials conducted were 10 per participant with 60 s assigned for the BCI data collection (similar to the first part) in order to stabilize the input data and 40 s (14 s for walking, limited by the walking path due to the optical fiber length constriction) assigned for FBG and BCI data collection for the second part of the experiment. It was ensured that the time stamp and initiation of signals for each activity, i.e., sitting, standing, and walking, matched for BCI and FBG. The sampling rate for both data streams was 125 Hz whereas the in-built capability of the Interrogator was 3 kHz and of the BCI was 250 Hz. The schematic for the experiment is shown in Figure 20, in addition to the BCI data collection as discussed in Section 4.1.1. The data from both FBG sensors and the BCI device were investigated for any outliers and/or artifacts that influence the results of the classification and prediction studies. The following provides detail on the instructions to participants while recording data in sitting, standing, and walking postures.

**Figure 21:**
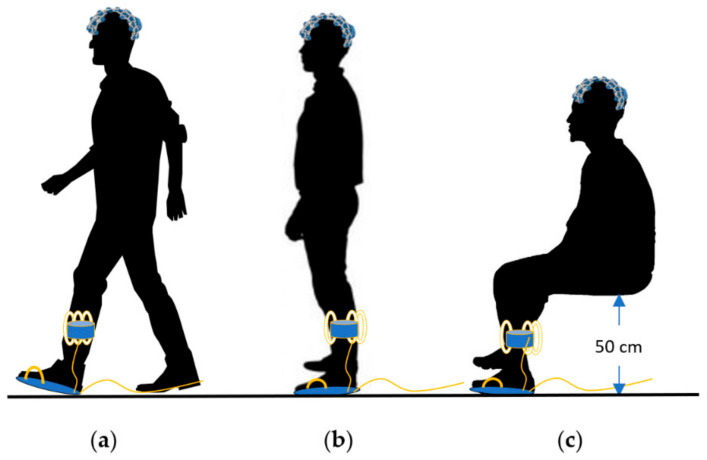
(**a**) Walking (**b**) standing (**c**) sitting postures of the participants while wearing equipment.

The participant sat in a chair of height 50 cm while wearing the BCI device correctly and resting his feet on a board where the sole is attached after taking off his shoes. This setup was designed to take the sitting data for 60 s for each of the four participants, each repeated 10 to 12 times for a total of 43 sitting data files. For the standing data, 60 s were recorded, and repeated 10 to 15 times for a total of 51 files. The walking data were recorded for 15 s repeated 20 times for each of the three participants for a total of 60 files, where the path allows for an average of 14 steps. Figure 21 illustrates (a) walking, (b) standing, and (c) sitting postures of the participants while wearing equipment.

**Figure 22:**
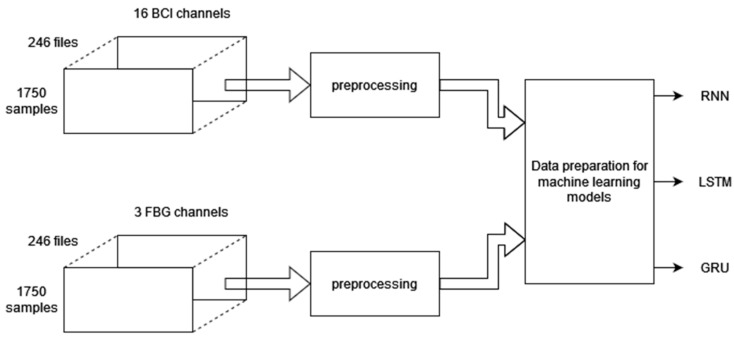
Signal flow diagram for the FBG-BCI machine learning model.

The data obtained from the BCI and FBG were cleaned and preprocessed for the final stage of correlation. A machine learning model fitted the 3 FBG signals to selected BCI channels chosen by reading the literature and the results of previous experiments on channel classification. After reducing the number of BCI channels and preprocessing both the BCI and FBG data, a neural network model was used to predict the BCI channel waveform using the three FBG signals. Three models were considered in this project: Recurrent Neural Network (RNN), Long Short-term Memory (LSTM), and Gated Recurrent Unit (GRU). In Figure 22, the signal flow diagram for the machine learning process is summarized.

#### 4.2.2. BCI Data Pre-Processing

For the 40 s sitting and standing data, the first and last 5 s were removed for stability purposes. Furthermore, the 30 s file was divided into two files of 15 s each to match the length of the 15 s walking data. However, after examining the walking data, another second from the beginning of each file had to be removed to keep a clean signal. The new dataset now was made up of 14 s files. The dataset now consisted of 84 sitting data files, 102 standing data files, and 60 walking data files. Wavelet decomposition was used to remove noise and signal trends represented by the wavelet approximation, and the first detail, “sym2” wavelet was used for the decomposition.

#### 4.2.3. FBG Data Pre-Processing

**Figure 23:**
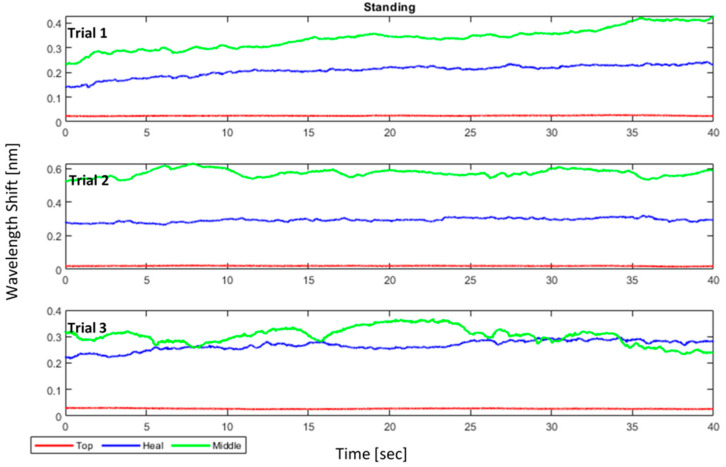
Standing posture wavelength-shifts for 3 trials of a single participant (before normalization).

For the FBG data, minimal preprocessing was required since the optical sensors do not pick up noise from the surroundings, but outliers had to be corrected as seen in Figure 23. Some outliers were caused by a sudden zero reading in the channel. In contrast, others were caused by the data from one channel being recorded in a different channel. The outliers were detected using the generalized extreme Studentized deviate test for outliers (gesd) and filled using shape-preserving piecewise cubic spline interpolation (pchip). Some files that had a large number of zeros had to be processed by the median detection method. After cleaning the data, the files were normalized by finding the maximum and minimum points of the three waveforms. While normalization is often applied as a part of data preparation for machine learning, its purpose is to change the values of numeric columns in the dataset to a common scale without distorting differences in the ranges of values. The data were normalized to [0, 1] using min-max scaling as also reported in [48].

To match the length of the BCI dataset, the first second from each file was also removed to form 14 s files matching the BCI data files. Finally, both FBG and BCI Raw, Wavelet, and Processed data were combined in one file with 1750 samples and a sampling rate of 125. After that, a total of 250 files for each data set were ready to be inserted into the machine learning model. 

The participants’ focus and immobility are important during collecting the data for the pressure profile. Since the FBG sensors are highly sensitive and may detect any movement, the recording is useless if the person is moving or not implementing the correct posture.

**Figure 24:**
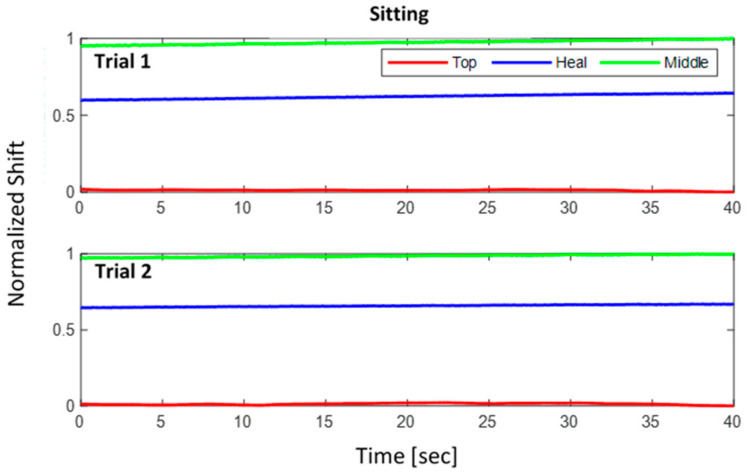
Sitting posture normalized shift for 2 trials of a single participant.

**Figure 25:**
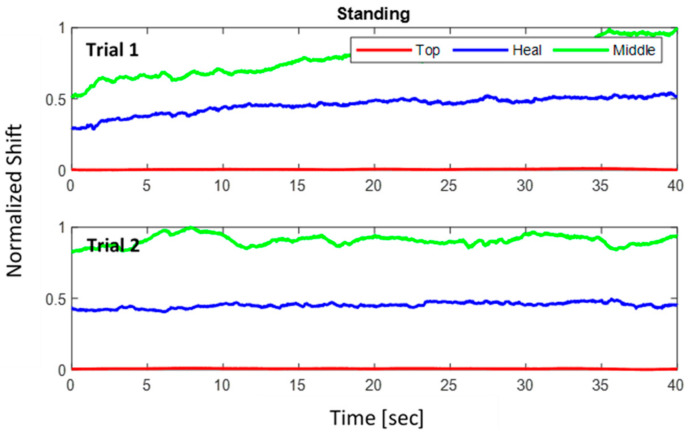
Standing posture normalized shift for 2 trials of a single participant.

**Figure 26:**
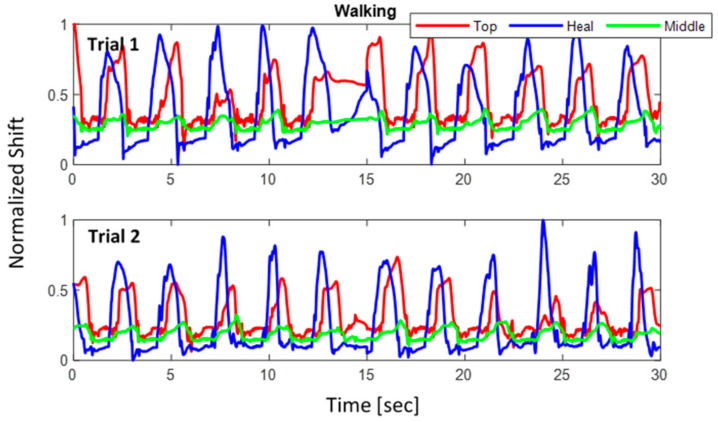
Walking posture normalized shift for 2 trials of a single participant.

However, there is no way to guarantee consistent results every time; as shown in Figure 23, the pressure data for the same person’s posture are obtained consecutively, yet some variances may be noticed. Furthermore, these data show similar behavior as expected, e.g., for standing, maximum pressure is on the middle of the foot, then on the heel, and lastly on the toes, which is the case for almost all the recorded data. However, the exact values can be different. These values represent the wavelength shift of the FBGs original wavelength, which is given in Equation (5) as:

(5) $$\Delta \lambda =\lambda_{orig}-\lambda_{meas}$$

where Δλ is wavelength shift, $\lambda_{orig}$ is the original FBG wavelength and $\lambda_{meas}$ is the measured wavelength of the FBG. This value is affected by many factors, for example, participant’s weight, FBG positions, surface material and specifications, and participant’s posture. To minimize the error of the machine learning model, all the collected data of the FBGs are normalized using Equation (6):

(6) $$\Delta \lambda_{norm}=\frac{\lambda_{meas}-min\;\left(of\;3\;waveforms\right)\;}{max\;\left(of\;3\;waveforms\right)-min\;\left(of\;3\;waveforms\right)}$$

where $\Delta \lambda_{norm}$ is the normalized wavelength shift. Figure 24, Figure 25, Figure 26 show the three normalized waveforms of sitting, standing, and walking, respectively for a single participant between two trials. The results show significant consistency after the normalization was applied. Additionally, many outliers were also corrected during the course of extracting FBG data.

#### 4.2.4. Performance Measure

In the second part of the experiment, deep learning algorithms were used to create models for brain activity electrodes. To optimize each model, a loss function was required to verify the accuracy of prediction. Hence, the mean square error (MSE) function was used to maximize the prediction accuracy of the models. The MSE function is given by Equation (7):

(7) $$\mathrm{MSE}=\frac{1}{n}\overset{n}{\underset{i=1}{\sum }}{\left(y_{i}-{\hat{y}}_{i}\right)}^{2}$$

where n is the number of samples, $y_{i}$ is the targeted value, and ${\hat{y}}_{i}$ is the predicted value. 

**Figure 27:**
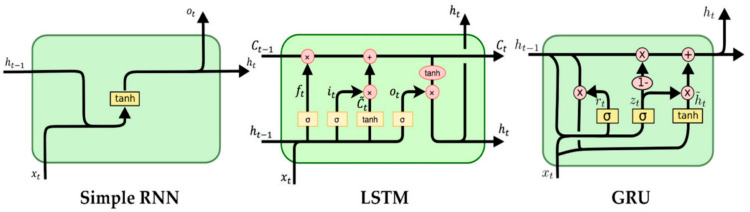
Schematics for the RNN, LSTM, and GRU machine learning models.

The deep learning algorithms built are RNN, LSTM, and GRU. The detailed schematics of the three algorithms are shown in Figure 27. All have been optimized with an Adam optimizer using the MSE loss function. The machine learning model considered data provided by three different types of processing methods: raw data, processed data, and wavelet data.

**Figure 28:**
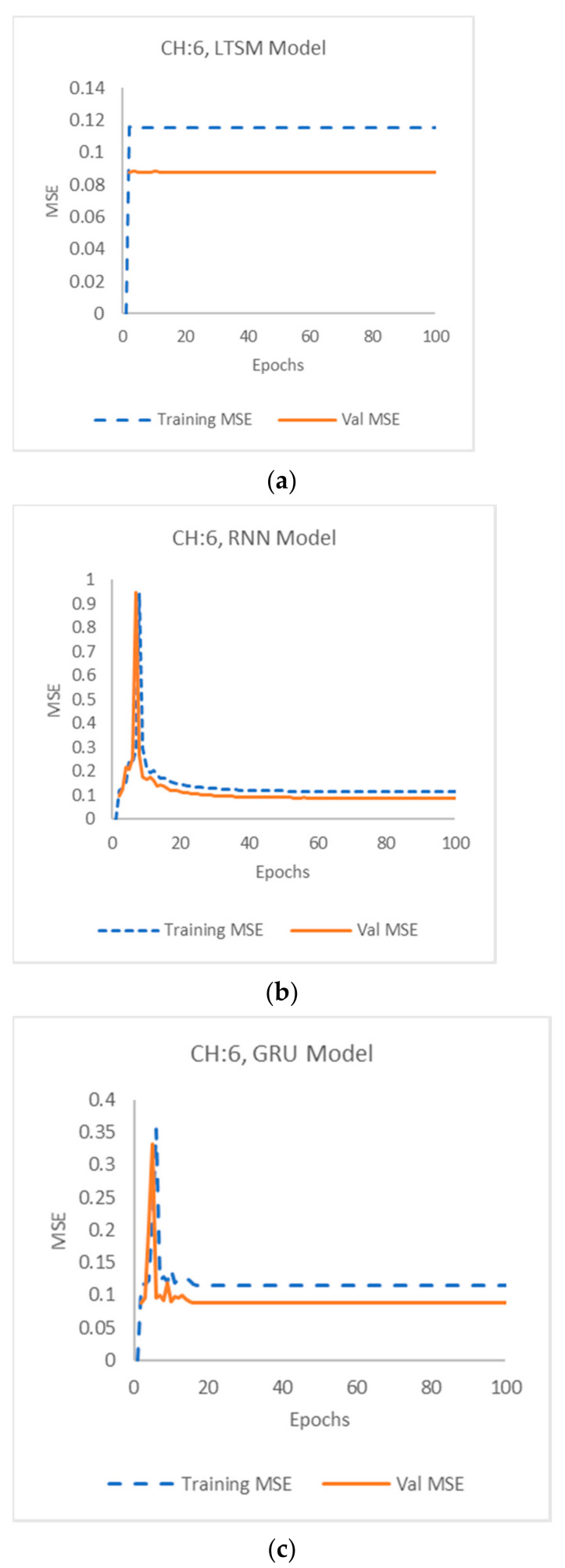
Channel 6: Training and validation error using (**a**) LSTM, (**b**) RNN and (**c**) GRU models.

**Figure 29:**
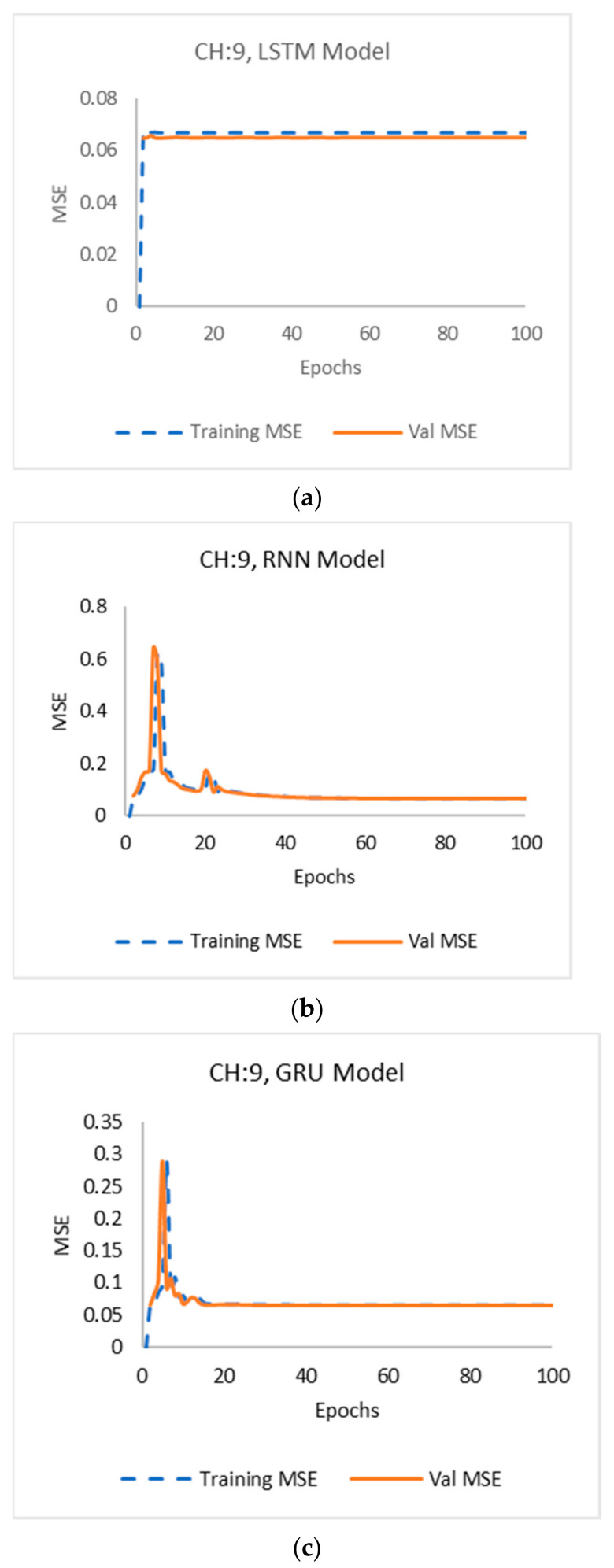
Channel 9: Training and validation error using (**a**) LSTM, (**b**) RNN and (**c**) GRU models.

Raw data are not recommended for use in most machine learning models, which was verified in this model by the extremely high training and validation MSE results they produced. Figure 28 shows the plot of the values of the training and validation MSE of LSTM, RNN, and GRU models, respectively, for the processed data in channel 6, whereas Figure 29 shows the plot of the values of the training and validation MSE of LSTM, RNN, and GRU models by wavelet decomposition, respectively, in channel 9.

**Figure 30:**
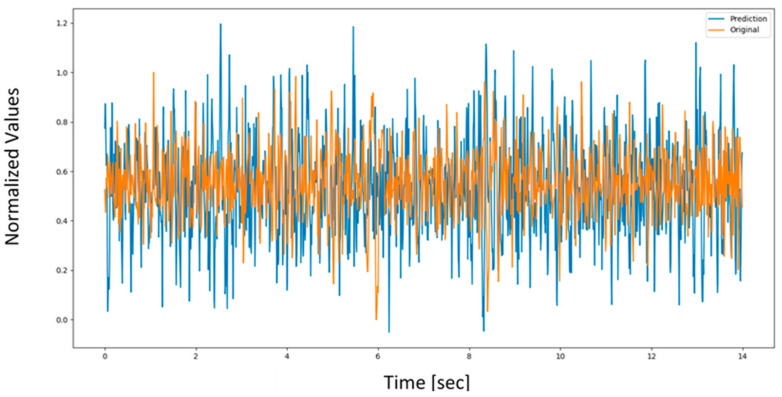
Channel 2 prediction based on the three FBG inputs for the LSTM model.

**Figure 31:**
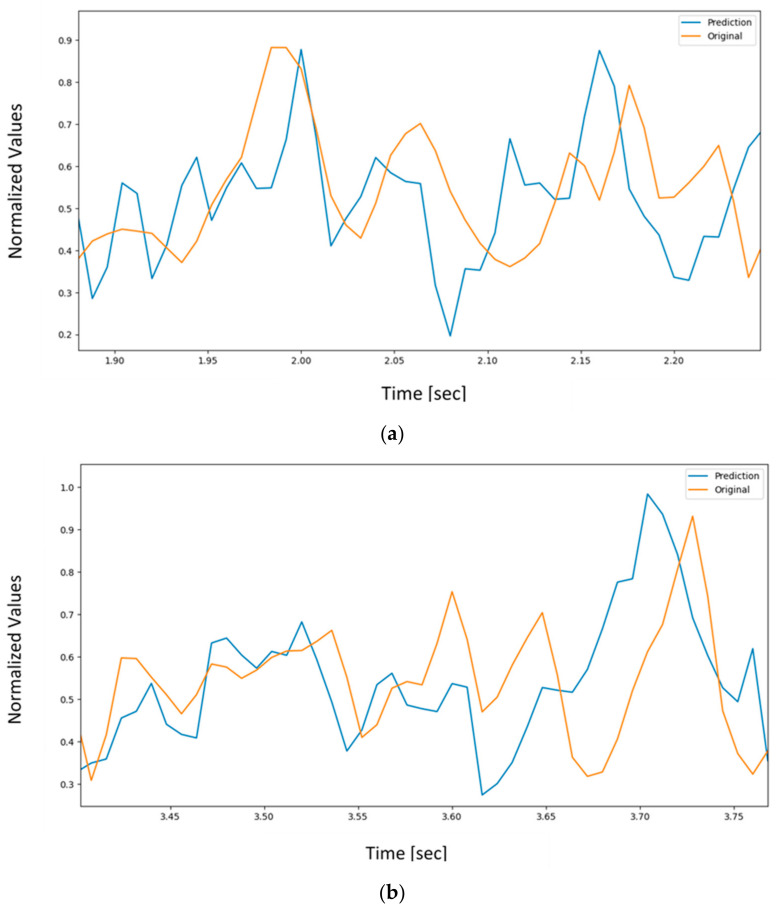
(**a**) A close-up of Figure 30 from 1.90 to 2.25 s; (**b**) close-up of Figure 30 from 3.4 to 3.8 s.

It was observed that the LSTM has the least error and requires the least number of epochs to reach a steady state. Figure 30 compares the original and predicted BCI signal at channel 2 as a sample based on unknown FBG data. Figure 31 provides a close-up at two instances to clearly show the resemblance between the original and the predicted signal.

## 5. Conclusions

A deep learning model that can predict brain activity signals for 6 electrodes of the 16 available from the BCI device using plantar pressure was built and tested. The deep learning algorithms built are RNN, LSTM, and GRU. All have been optimized using the Adam optimizer with MSE loss function. The raw data showed the worst model performance, as expected from the literature on deep learning performance. The processed data (normalized, detrended, filtered) performed well with a minimum MSE of 0.0877 for channel 12. The wavelet decomposed data have the best overall performance with 0.0757, 0.0919, 0.1093, 0.065, 0.784, and 0.093 MSE values for electrodes 2, 5, 6, 9, 11, and 12, respectively. The wavelet decomposed data have lower MSE for all electrodes, except electrode 12. 

The six electrodes were chosen based on classification models, in which BCI data were collected for three posture positions (sitting, standing, and walking). In the classification models, the BCI data were organized by channel. Hence, there is a classifier for every channel for each classification algorithm (K-NN, SVM, logistic regression, and naïve Bayes). Furthermore, the classifiers were tested on raw data and processed data (normalized, detrended, and filtered). The best performing posture classifiers are for channel 2, 5, 6, 9, 11, and 12 with accuracy of 92%, 81%, 85%, 93%, 92%, and 87%, respectively. Moreover, the plantar pressure was obtained using three FBG sensors on the maximum pressure points in the foot. 

While the final aim of the research was to predict brain signals from given plantar pressure information, the classification accuracy played an important role. The classification accuracy of above 80% when compared to previous works as cited in a comprehensive review [49] provided confidence in the results. However, for future work a thorough investigation is suggested with a combination of different numbers of electrodes and FBG sensors to identify the trend in increasing/decreasing accuracy of results in classification and prediction.

## 6. Recommendations

1. The FBG optical cables can be replaced with free-space optical communications to obtain the data wirelessly and allow longer distance and duration for trial.
2. Deep learning algorithms can be written from scratch to have lower MSE instead of using PyTorch models.
3. Different types of foot insoles can be used to obtain a variety of plantar pressure data.
4. Adding a temperature element in the insole to examine the effect of plantar temperature on brain activity signals.
5. For production, the current BCI device can be substituted by a BCI device with a few channels, which will cut costs and make this process cheaper and more accessible.
6. Since the FBG sensors are fragile, the insole should have a grooved housing for the sensors’ protection, with a hard material to preserve the pressure sensitivity. The FBG length should also be shortened to extend only to the ankle and connected to a portable light source and monitor. It will significantly enhance mobility and allow for more accurate testing.
7. Furthermore, if the machine learning model had more data to learn from, a more robust trained model could be developed for medical and research use.
8. Further research into miniature embeddable sensors such as FBGs will allow exploring their potential for providing a sensorial base to mimic the human sense of touch.

## Figures and Tables

**Table 1:** Information of participants for the study.

Participant	Age	Weight (kg)	Height (m)	Foot Size (EU)
1	23	102	1.73	42.5
2	24	67	1.77	42
3	23	72	1.80	43
4	23	70	1.70	41

**Table 2:** Accuracy results of channel classification.

	SAMPLED																PROCESSED															
	K-NN				SVM				Logistic Regression				Naïve Bayes				K-NN				SVM				Logistic Regression				Naïve Bayes			
CH	TR	VAL	T	BT	TR	VAL	T	BT	TR	VAL	T	BT	TR	VAL	T	BT	TR	VAL	T	BT	TR	VAL	T	BT	TR	VAL	T	BT	TR	VAL	T	BT
1	1	0.81	0.75	0.8	0.87	0.83	0.75	0.8	1	0.59	0.33	0.37	0.72	0.73	0.58	0.5	1	0.51	0.33	0.36	1	0.41	0.5	0.56	1	0.38	0.25	0.29	0.81	0.56	0.67	0.69
2	0.74	0.74	0.92	0.92	0.83	0.78	0.67	0.61	1	0.55	0.25	0.24	0.74	0.72	0.66	0.61	1	0.45	0.17	0.22	1	0.43	0.25	0.29	1	0.45	0.25	0.25	0.68	0.55	0.58	0.6
3	1	0.68	0.5	0.54	0.78	0.78	0.42	0.4	1	0.49	0.42	0.44	0.7	0.66	0.58	0.62	1	0.51	0.5	0.58	1	0.44	0.42	0.5	1	0.38	0.25	0.33	0.66	0.51	0.58	0.6
4	1	0.59	0.75	0.78	0.98	0.73	0.75	0.69	1	0.43	0.33	0.32	0.55	0.53	0.5	0.51	1	0.49	0.67	0.67	1	0.32	0.67	0.72	1	0.25	0.75	0.78	0.68	0.62	0.75	0.67
5	0.81	0.77	0.83	**0.81**	1	0.28	0.5	0.54	1	0.49	0.33	0.32	0.68	0.68	0.67	0.67	1	0.38	0.58	0.53	1	0.36	0.67	0.69	1	0.32	0.75	0.76	0.62	0.47	0.75	0.75
6	1	0.53	0.83	**0.85**	1	0.42	0.42	0.5	1	0.45	0.33	0.36	0.51	0.51	0.67	0.6	1	0.51	0.33	0.42	1	0.47	0.5	0.58	1	0.4	0.42	0.47	0.74	0.55	0.83	**0.85**
7	1	0.62	0.58	0.59	1	0.36	0.33	0.37	1	0.49	0.33	0.33	0.64	0.6	0.67	0.64	1	0.45	0.33	0.42	1	0.44	0.25	0.29	1	0.44	0.33	0.37	0.83	0.55	0.33	0.31
8	1	0.51	0.67	0.67	1	0.42	0.42	0.47	1	0.38	0.25	0.28	0.49	0.49	0.33	0.32	1	0.49	0.25	0.33	1	0.34	0.42	0.47	1	0.32	0.42	0.5	0.83	0.64	0.5	0.48
9	1	0.6	0.92	**0.93**	1	0.33	0.25	0.3	1	0.52	0.58	0.56	0.55	0.53	0.58	0.53	1	0.49	0.25	0.24	1	0.32	0.25	0.28	1	0.29	0.16	0.19	0.74	0.53	0.92	**0.92**
10	1	0.54	0.58	0.59	1	0.25	0.33	0.42	1	0.62	0.33	0.39	0.55	0.56	0.58	0.61	1	0.42	0.25	0.33	1	0.3	0.25	0.33	1	0.27	0.33	0.4	0.79	0.59	0.42	0.48
11	1	0.54	0.5	0.43	1	0.33	0.5	0.57	1	0.32	0.33	0.36	0.55	0.54	0.67	0.68	1	0.47	0.42	0.5	1	0.45	0.58	0.65	1	0.29	0.42	0.46	0.7	0.53	0.92	**0.92**
12	0.74	0.7	0.5	0.51	1	0.47	0.5	0.58	1	0.29	0.33	0.36	0.57	0.6	0.42	0.4	1	0.56	0.5	0.58	1	0.51	0.5	0.58	1	0.42	0.5	0.58	0.85	0.7	0.83	**0.87**
13	1	0.47	0.42	0.47	1	0.33	0.25	0.33	1	0.4	0.33	0.39	0.51	0.48	0.33	0.33	1	0.45	0.5	0.57	1	0.36	0.42	0.47	1	0.34	0.33	0.39	0.55	0.43	0.42	0.43
14	1	0.55	0.67	0.67	1	0.28	0.33	0.4	1	0.3	0.08	0.07	0.51	0.38	0.33	0.33	1	0.43	0.42	0.48	1	0.23	0.33	0.39	0.96	0.28	0.58	0.67	0.83	0.45	0.33	0.36
15	1	0.69	0.67	0.63	1	0.42	0.33	0.42	1	0.42	0.33	0.33	0.57	0.55	0.42	0.4	1	0.43	0.58	0.66	1	0.32	0.5	0.58	1	0.28	0.42	0.5	0.8	0.49	0.5	0.51
16	1	0.59	0.5	0.51	1	0.44	0.42	0.47	1	0.48	0.33	0.33	0.32	0.73	0.42	0.33	1	0.38	0.42	0.47	1	0.41	0.42	0.47	1	0.34	0.25	0.3	0.77	0.51	0.5	0.54
Avg	0.96	0.621	**0.662**	**0.669**	0.97	0.47	0.45	0.49	1	0.45	0.33	0.34	0.57	0.58	0.53	0.51	1	0.46	0.41	0.46	1	0.38	0.43	0.49	1	0.34	0.4	0.45	0.74	0.54	**0.61**	**0.62**

TR: Training accuracy; VAL: Validation Accuracy; T: Test Accuracy; BT: TEST Balanced Accuracy.

## Data Availability

The data presented in this study are available on request from the corresponding author. The data are not publicly available due to prior consent from the institution/funding agency.
